# Diagnosis and Management of Preeclampsia: Suggested Guidance on the Use of Biomarkers

**DOI:** 10.1055/s-0042-1744286

**Published:** 2022-04-25

**Authors:** Maria Laura Costa, Ricardo de Carvalho Cavalli, Henri Augusto Korkes, Edson Vieira da Cunha Filho, José Carlos Peraçoli

**Affiliations:** 1 Department of Gynecology and Obstetrics, Universidade Estadual de Campinas, Campinas, SP, Brazil; 2 Department of Gynecology and Obstetrics, Faculdade de Medicina de Ribeirão Preto, Universidade de São Paulo, Ribeirão Preto, SP, Brazil; 3 Department of Human Reproduction and Childhood, Pontifícia Universidade Católica de São Paulo, São Paulo, SP, Brazil; 4 Obstetrics and Gynecology Department, Hospital Moinhos de Vento-HMV, Porto Alegre, RS, Brazil; 5 Department of Gynecology and Obstetrics, Faculdade de Medicina de Botucatu, Universidade Estadual Paulista Júlio de Mesquita Filho, Botucatu, SP, Brazil

**Keywords:** preeclampsia, hypertension, placental growth factor, preterm preeclampsia, soluble fms-like tyrosine kinase 1, pré-eclâmpsia, hipertensão, fator de crescimento placentário, pré-eclâmpsia prematura, tirosina quinase 1 tipo fms solúvel

## Abstract

**Objective** It is a challenge to consider preeclampsia (PE) diagnosis and management in low and middle-income settings, where it represents a major public health concern. The placenta is the underlying cause of disease, and the plasma concentrations of proangiogenic and antiangiogenic factors released by the placenta can reflect the risks of disease progression. Antiangiogenic proteins, such as soluble fms-like tyrosine kinase 1 (sFlt-1), and proangiogenic, like placental growth factors (PlGF), are directly and inversely correlated with the disease onset, respectively.

**Methods** Narrative review on the use of biomarkers (sFlt-1 to PlGF ratio) with a suggested guidance protocol.

**Results** Key considerations on the use of biomarkers: the sFlt-1/PlGF ratio is mainly relevant to rule out PE between 20 and 36 6/7 weeks in cases of suspected PE; however, it should not replace the routine exams for the diagnosis of PE. The sFlt-1/PlGF ratio should not be performed after confirmed PE diagnosis (only in research settings). In women with suspected PE, sFlt-1/PlGF ratio < 38 can rule out the diagnosis of PE for 1 week (VPN = 99.3) and up to 4 weeks (VPN= 94.3); sFlt-1/PlGF ratio > 38 does not confirm the diagnosis of PE; however, it can assist clinical management. In cases of severe hypertension and/or symptoms (imminent eclampsia), hospitalization is imperative, regardless of the result of the sFlt-1/PlGF ratio.

**Conclusion** The use of biomarkers can help support clinical decisions on the management of suspected PE cases, especially to rule out PE diagnosis, thus avoiding unnecessary interventions, especially hospitalizations and elective prematurity

## Resumo

**Objetivo** É um desafio considerar o diagnóstico e o tratamento da pré-eclâmpsia (PE) em locais de baixa e média renda, onde a doença representa um grande problema de saúde pública. A placenta é a causa subjacente da doença, e as concentrações plasmáticas de fatores pró-angiogênicos e antiangiogênicos liberados pela placenta podem refletir os riscos de progressão da doença. Proteínas antiangiogênicas, como a tirosina quinase fms solúvel tipo 1 (sFlt-1), e pró-angiogênicas, como o fator de crescimento placentário (PlGF), estão direta e inversamente correlacionados com o início da doença, respectivamente.

**Métodos** Revisão narrativa sobre o uso de biomarcadores (razão sFlt-1/PlGF) com sugestão de protocolo de orientação para uso clínico.

**Resultados** Principais considerações sobre o uso de biomarcadores: a razão sFlt-1/PlGF é principalmente relevante para descartar PE entre 20 e 36 6/7 semanas em casos de suspeita de PE; entretanto, não deve substituir os exames de rotina para o diagnóstico de PE. A relação sFlt-1/PlGF não deve ser realizada após a confirmação do diagnóstico de PE (apenas em ambientes de pesquisa). Em mulheres com suspeita de PE, a razão sFlt-1/PlGF < 38 pode descartar o diagnóstico de PE por 1 semana (VPN = 99,3) e até 4 semanas (VPN = 94,3); A relação sFlt-1/PlGF > 38 pode auxiliar no manejo clínico. Em casos de hipertensão grave e/ou sintomas (eclâmpsia iminente), a hospitalização é imprescindível, independentemente do resultado da relação sFlt-1/PlGF.

**Conclusão** O uso de biomarcadores pode auxiliar na tomada de decisões clínicas no manejo de casos suspeitos de PE, principalmente para afastar o diagnóstico da doença, evitando intervenções desnecessárias, tais como internações e prematuridade iatrogênica.

## Introduction

**Fig. 1:**
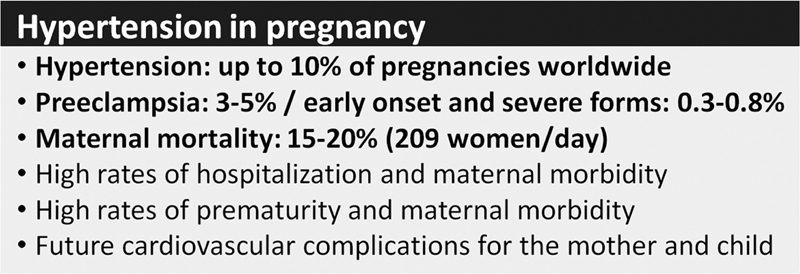
Summary of the overall impact of hypertension in preeclampsia.

It is a great challenge to consider preeclampsia (PE) diagnosis and management in settings of low and middle-income, where the burden of the disease still represents a major public health concern, with high impact ( Fig. 1 ) in maternal mortality and morbidity. 1 2 Even with considerable advances in research and healthcare, the management of PE has changed little in the last decades, with outcomes relying on accurate diagnosis, identification of severity and decision on the timing of delivery. 3 Globally, 42,000 women die each year from PE, and, for each death, other 50 to 100 women suffer from considerable morbidity. 2 4

There are few excellent examples of success in reducing maternal mortality due to hypertensive disorders, such as the United Kingdom (UK). Over the past 65 years, UK presented an expressive drop in avoidable direct causes of maternal mortality, with fewer than 1 in 10,000 deaths among pregnant and postpartum women currently. 5 During the last report, with data from 2012 to 2014, UK had only two maternal deaths due to hypertensive disorders. 6 If we compare with the same period in Brazil, the reported number of maternal deaths due to hypertension was a shocking 971 women. 7

The investment in national guidelines and recommendations for clinical care, service organization, and research priorities have been highlighted as responsible for such results in the UK, with improved surveillance, diagnosis, and timely delivery. Special focus on severe cases, including pulmonary edema, with fluid restriction protocols and intracerebral hemorrhage, with adequate treatment of severe hypertension are examples of targeted and effective recent interventions. Identifying conditions involved in the decrease in maternal deaths from hypertensive disorders in the UK should help other health systems to reduce their maternal death rates. 5 6 8

Preeclampsia is certainly one of the most challenging situations during pregnancy. It is not only influenced by innumerous conditions (genetic, immunological, environmental) but it can also affect all organs, however in different ways, and we never know ahead of time which patient will present with what symptoms and complications. For example, a patient can be identified in a routine assessment with classic hypertension and proteinuria, while others can open with seizures or severe placental compromise, with fetal growth restriction or even placental abruption. 3 Knowing that, and with the growing understanding of the role the placenta plays, especially in early onset PE (< 34 weeks gestation), in the last decades, studies have advanced in showing that plasma concentrations of proangiogenic/ antiangiogenic factors, released by the placenta (syncytiotrophoblast) can reflect the risks of disease progression. Antiangiogenic, proteins such as soluble fms-like tyrosinekinase 1 (sFlt-1), and proangiogenic placental growth factors (PlGF) directly and inversely correlate, respectively, with disease onset. 9

The updated UK national guideline, National Institute for Health and Care Excellence (NICE), has included, for women with suspected PE, a recommendation that says: “triage PlGF test and the Elecsys immunoassay sFlt-1/PlGF ratio, used with standard clinical assessment and subsequent clinical follow-up, are recommended to help rule out PE in women presenting with suspected PE between 20 weeks and 34 weeks plus 6 days of gestation.” 10 Other international guidelines have also incorporated plasma concentrations of proangiogenic/antiangiogenic factors in their recommendations. 11 12 Recently, these exams were authorized by the Brazilian National Agency for Supplementary Health (ANS). However, in a setting with such a high impact of the condition, we believe that the use of biomarkers must be supported by guidelines and that it must be in accordance with national protocols on diagnosis and management of preeclampsia. 13 We present a suggestion for such implementation, considering key measurement cutoff points that have been recently identified to have a high negative predictive value for PE.

## Relevant Definitions Considering PE

**Fig. 2:**
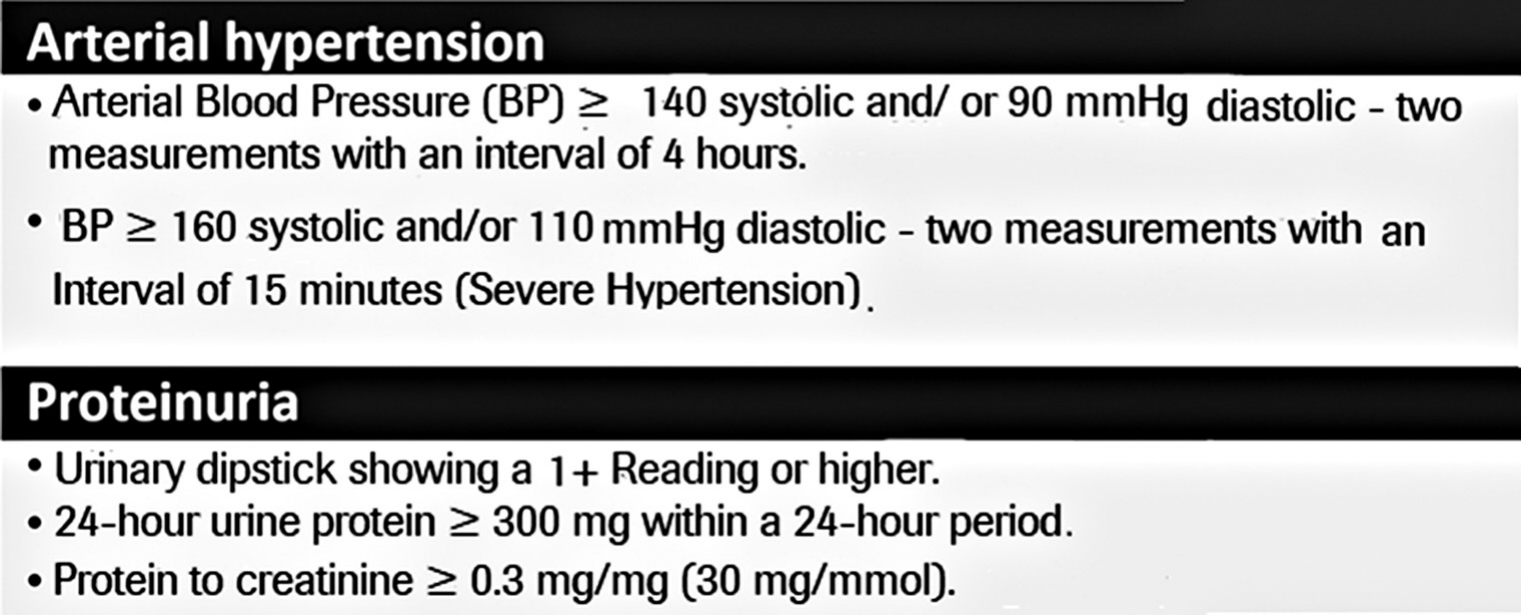
Definition of arterial hypertension and proteinuria.

**Fig. 3:**
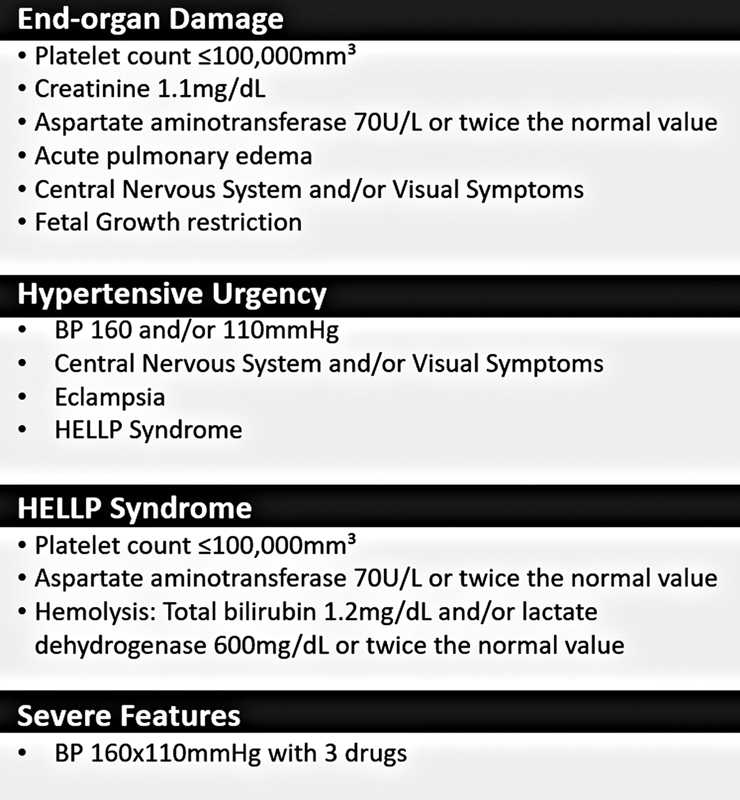
End-organ damage and severe disease.

A major concern is the adequate diagnosis of the condition and severity. Definitions of hypertension, proteinuria, end-organ damage, and severe disease are listed in the boxes below ( Figs. 2 and 3 ). Preeclampsia is considered when hypertension arises in previous normotensives women, after 20 weeks gestation, with proteinuria. In the absence of proteinuria, if there are signs of severity or end-organ damage, the diagnosis is also confirmed. 13

### When and How to Consider the Use of Angiogenic and/or Antiangiogenic Factors

**Fig. 4:**
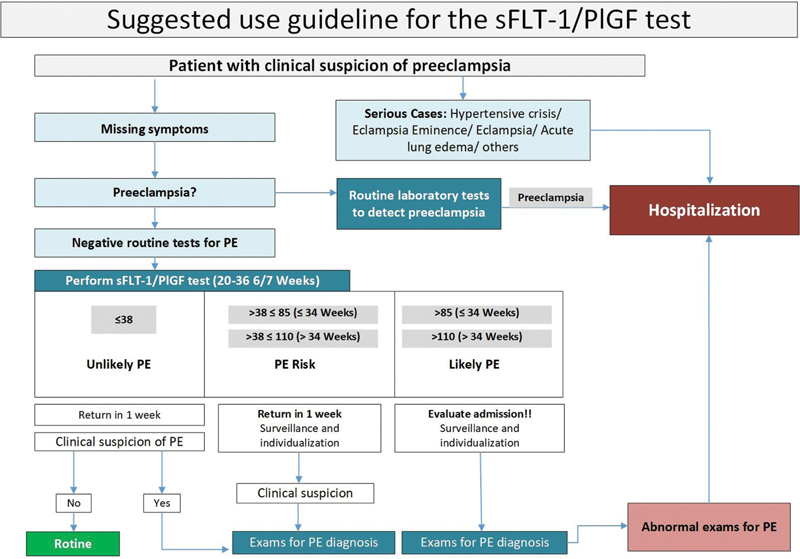
Suggestion for using the sFlt-1/PlGF ratio in clinical practice.

Figure 4 presents a suggested guideline/flowchart toward a suspected case of PE and situations that could benefit from testing for proangiogenic and/or antiangiogenic factors. Considering women with clinical suspicion of PE, we must be careful to carry out confirmatory tests. However, in cases with severe features, emergency assistance must be imperative. Patients who present with severe hypertension (systolic BP ≥ 160 and/or diastolic BP ≥ 110 mm Hg), symptoms suggestive of imminent eclampsia (headache, scotomas and/or epigastric pain), acute pulmonary edema, elevation of liver enzymes, thrombocytopenia, among others, should receive immediate assistance and admission to a referral center. Under no circumstances should the sFlt-1/PlGF test delay or guide approaches in these cases. 10 13

Considering women with clinical suspicion of PE, without severe features, it is important to perform tests toward the proper diagnosis. After PE confirmation by the known recommended tests, the patient should be referred to a referral center for adequate follow-up (depending on gestational age and findings). The sFlt-1/PlGF ratio does not replace the usual tests for the diagnosis of PE and, at this time, should not be performed for diagnostic confirmation.

Women with clinical suspicion of PE, who present negative tests for the diagnosis of PE (absence of proteinuria or target organ damage) between 20 and 36 6/7 weeks, should undergo the sFlt-1/PlGF test. In these cases, the sFlt-1/PlGF ratio can help in more adequate follow-up and care planning. 10

- sFlt-1/PlGF ≤ 38

The sFlt-1/PLGF ratio at a threshold of 38 can reassure about the absence of PE at that giving time, as well as indicate a low possibility of onset in the following week, with a negative predictive value (NPV) of 99.3% (97.9–99.9). 14 It can also help in the clinical reasoning regarding the non-appearance of PE in 2 weeks [NPV 97.9% (96.0–99.0)], 3 weeks [NPV 95 0.7% (93.3–97.5)], and up to 4 weeks [NPV 94.3% (91.7–96.3)]. 15 Nevertheless, even with low values of the sFlt-1/PLGF ratio, given a new clinical suspicion of PE, due to suggestive signs and/or symptoms, the team should proceed with PE investigation through routine exams.

- sFlt-1/PlGF > 38 and ≤ 85 (≤ 34 weeks) and >38 and < 110 (> 34 weeks)

Values above 38, but below 85 up to 34 weeks or below 110 after 34 weeks indicate a higher risk of PE. However, due to the low positive predictive value (PPV) in this situation [PPV 36.7% (28.4–45.7)], the diagnosis of PE cannot be ascertained exclusively by the ratio. 14 These patients need close surveillance for maternal and fetal assessment and new PE investigation depending on clinical findings. Medical visits should be frequent, with adequate counseling on possible suggestive signs and symptoms of severe features.

- sFlt-1/PlGF > 85 (≤ 34 weeks) and > 110 (> 34 weeks)

These higher ratio values, although not confirmatory of PE, may reflect, in clinical practice, conditions associated with increased risks of adverse outcomes. The management of such cases will depend on the local institutional protocol, but greater maternal and fetal surveillance in this group of patients is necessary. In individual cases and in accordance with local protocols, such patients may be hospitalized for closer follow-up. Again, when there is a clinical suspicion of PE or severe features, the usual tests for its diagnosis should always be performed.

## Cost-effectiveness of Biomarker Testing

Considering cost-effectiveness, some studies, conducted in different countries have shown that the use of sFlt-1/PlGF tests compared with non-use to manage patients with suspected PE, could be cost-saving, by avoiding unnecessary procedures and hospitalization. Analysis of the economic impact in the UK indicated that hospitalizations of women with suspected preeclampsia were reduced by 56%, resulting in savings of £344 per patient. 16 17 18 19 The cost-saving per patient was also found in other countries, such as Italy, Germany, Switzerland (ranging between € 346 and €670) (17–19), US ($1,215), 20 and Japan (16,373 JPY). 21 In Brazil, comparing public and private health care, the calculated savings was R$185.06 and R$635.84 per patient, respectively. 22

In another study, a probability model was assessed to verify the PlGF testing cost-effectiveness. The use of PlGF testing for suspected preterm PE had a 59.9% probability of representing a cost-saving compared with the current practice, with a total cost-saving of £149 per woman when including the cost of the test. Given the estimated number of births in England and the incidence of pregnant women that have suspected PE before 37 weeks, PlGF testing could result in a potential cost-saving of £2,891,196 each year across the English NHS. The majority of cost-savings associated with PlGF testing are through a reduction observed in maternal outpatient appointments among women testing with a PlGF > 100 pg/ml. 23 24

Although the cost-analysis to rule out PE seems to reduce expenses, more evidence is needed when considering such intervention in clinical practice and especially in low and middle-income settings.

1. The sFlt-1/PLGF ratio is an ally in the diagnosis of PE, mainly to rule out suspected cases among patients with clinical suspicion of PE between 20 and 36 6/7 weeks;
2. The test should not be performed alone, in the first half of pregnancy, for the early prediction of PE;
3. In suspected cases of PE, the sFlt-1/PlGF ratio can be requested; however, it should NOT replace the routine exams for the diagnosis of PE, which should be mandatory;
4. The sFlt-1/PlGF ratio should NOT be performed after a confirmed PE diagnosis. This use finds support in research settings but not in clinical practice;
5. The sFlt-1/PlGF ratio should NOT be performed routinely in patients with no clinical suspicion of PE, as a screening for the disease;
6. In women with suspected PE, a sFlt-1/PlGF ratio < 38 can rule out the diagnosis of PE for 1 week (VPN = 99.3) and up to 4 weeks (VPN= 94.3);
7. In women with suspected PE, a sFlt-1/PlGF ratio > 38 does not confirm the diagnosis of PE; however, it can assist clinical management;
8. In cases of severe hypertension (BP ≥ 160 and/or PAd ≥ 110 mm Hg) and/or symptoms (imminent eclampsia), hospitalization is imperative, regardless of the result of the sFlt/PlGF ratio;
9. The sFlt/PlGF ratio should NOT be requested every week (re-test) in cases that do not present again a clinical suspicion of PE;
10. The sFlt-1/PlGF ratio should NOT be used to define timing of delivery.

## Conclusion

The use of biomarkers in obstetric clinical practice is a reality in many countries and can help support clinical decisions on the management of cases, enabling more accurate differential diagnoses and, mostly, excluding the diagnosis of PE, thus avoiding unnecessary interventions, especially hospitalizations and elective prematurity (10). However, to ensure adequate use of biomarkers, it is key to follow a protocol that considers clinical findings and interpretation of results.
